# U-shaped association between the non-high-density lipoprotein cholesterol to high-density lipoprotein cholesterol ratio and mortality risk in obese adults: evidence from NHANES 1999–2018

**DOI:** 10.3389/fcvm.2024.1524465

**Published:** 2025-01-10

**Authors:** Zi Lin, Tao Yi, Feng Hu, Jinhua Chen, Lianglong Chen

**Affiliations:** ^1^ Department of Cardiology, Fujian Medical University Union Hospital, Fuzhou, China; ^2^ Fujian Provincial Cardiovascular Medical Center, Fuzhou, China; ^3^ Fujian Provincial Coronary Heart Disease Research Institute, Fuzhou, China

**Keywords:** obesity, Non-HDL-C/HDL-C, American adults, all-cause mortality, cardiovascular mortality

## Abstract

**Background:**

Obesity, often accompanied by dyslipidemia and increased cardiovascular risk, poses a significant threat to overall mortality. The non-high-density lipoprotein cholesterol to high-density lipoprotein cholesterol ratio (NHHR) has been identified as a valuable parameter for assessing dyslipidemia. The goal of the study was to elucidate the relationship between NHHR and mortality in obese populations.

**Methods:**

Data for the study cohort were sourced from the National Health and Nutrition Examination Survey (1999–2018). The association between NHHR and mortality from all causes and cardiovascular disease was examined through multivariable Cox regression and restricted cubic splines (RCS). Segmented multivariable Cox regression and subgroup analyses were conducted when segmented effects were identified. The reliability of the results was confirmed through multiple sensitivity analyses.

**Results:**

A total of 7,504 participants were included in the analysis. During a median follow-up of 119 months, 866 subjects died for all causes, of which 318 were related to cardiovascular diseases. A U-shaped association was found utilizing RCS analysis, with cardiovascular mortality and all-cause mortality exhibiting the lowest risk points at 3.409 and 3.369, respectively. The fully adjusted model revealed a negative relationship between the risk of cardiovascular mortality (HR = 0.68, 95% CI: 0.49–0.94) and all-cause mortality (HR = 0.82, 95% CI: 0.67–1.00) for per 1 mmol/L increase in NHHR levels below the cut-off value. On the other hand, above the cut-off point, NHHR was positively correlated with cardiovascular mortality (HR = 1.18, 95% CI: 1.02–1.36) and all-cause mortality (HR = 1.13, 95% CI: 1.01–1.28). The sensitivity results of this study were in accordance with earlier findings, and no significant interactions in NHHR levels were discovered across different subgroups.

**Conclusions:**

In the obese adults, NHHR displayed a U-shaped relationship with cardiovascular and all-cause death. Monitoring and managing NHHR levels in obese population may help mitigate the risk of mortality.

## Introduction

Obesity is a chronic disease recognized as a global epidemic, affecting nearly 1 billion adults worldwide, including over 40% of Americans, and its prevalence continues to rise (1, 2). Obesity significantly elevates the risk of numerous cardiovascular diseases (CVD), which are the leading cause of mortality among obese individuals. CVD in this population substantially contributes to heightened rates of mortality and disability (3–5).

Existing studies have established a close association between dyslipidemia and obesity (6). In obese populations, adiposopathic dyslipidemia (or “atherogenic dyslipidemia”) is characterized by elevated serum triglycerides (TG), reduced high-density lipoprotein cholesterol (HDL-C), increased non-high-density lipoprotein cholesterol (Non-HDL-C), and the presence of small, dense low-density lipoprotein (sdLDL) particles (7). Dyslipidemia represents a critical pathway linking obesity to metabolic syndrome (MetS), CVD and various cancers (8). Therefore, appropriate lipid assessment indicators are vital for reducing cardiovascular and all-cause mortality in obese individuals.

The Non-HDL-C to HDL-C ratio (NHHR) has emerged as an innovative and comprehensive indicator for assessing atherogenic risk, as it simultaneously captures both atherogenic and anti-atherogenic lipid particles. NHHR has demonstrated significant associations with metabolic syndrome (9), type 2 diabetes (10), and atherosclerotic CVD (11, 12). Recent research has revealed a U-shaped association between NHHR and all-cause mortality in diabetic and prediabetic populations, while showing an L-shaped relationship with cardiovascular mortality (13).

NHHR may be particularly valuable in obese populations for several reasons. First, while LDL-C remains the primary atherogenic lipoprotein, it alone may not adequately reflect the full spectrum of cardiovascular risk in obese individuals, who typically present with increased triglyceride-rich lipoproteins and excess sdLDL particles (14, 15). Second, Non-HDL-C, which encompasses cholesterol from LDL, VLDL, IDL, and Lp(a) particles (17, 18), offers practical advantages including simpler calculation and greater stability regardless of TG levels or feeding status (16). Third, the consistently lower HDL-C levels observed in obese individuals, combined with its inverse correlation with cardiovascular risk (19), make the ratio particularly relevant for risk assessment in this population.

Despite these theoretical advantages, there was limited research examining the relationship between NHHR and mortality risk specifically in obese populations. Using the NHANES longitudinal cohort data from 1999 to 2018, this study aimed to investigate the association between NHHR and mortality in obese individuals and determine optimal NHHR thresholds for risk prediction. Our findings could inform targeted prevention and treatment strategies for this high-risk population.

## Materials and methods

### Study population

This study aimed to explore the predictive significance of NHHR for all-cause and cardiovascular mortality, attempting to seek possible threshold point. The civilian, non-institutionalized U.S. population's health and nutritional status are evaluated through the NHANES survey program. It consists of physical examinations conducted in mobile examination services as well as interviews conducted in homes (20).

**Figure 1:**
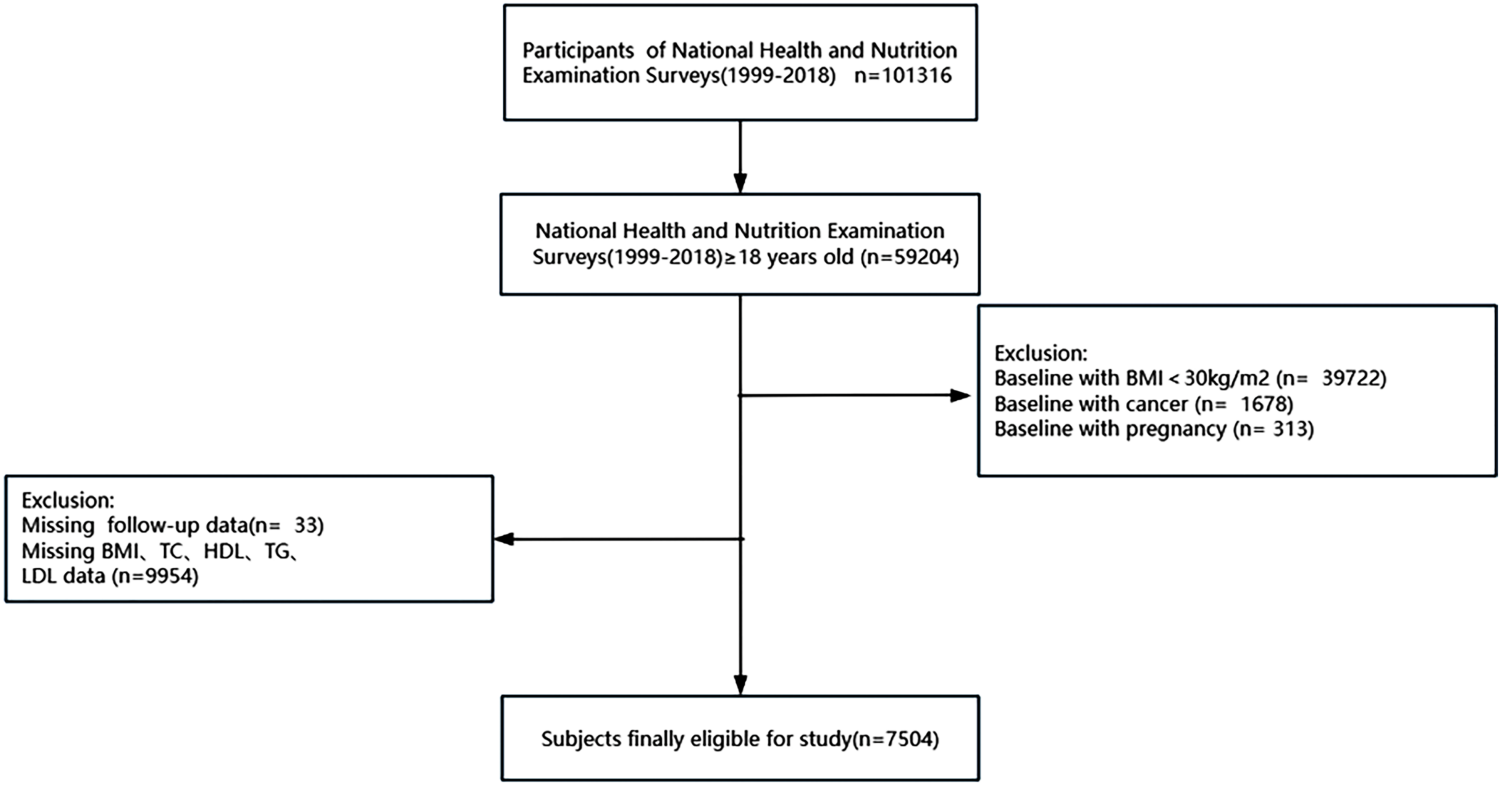
Participants recruitment and screening flowchart. BMI, body mass index; HDL-C, high-density lipoprotein cholesterol; LDL-C, low-density lipoprotein cholesterol; TC, total cholesterol; and TG, triglyceride.

A BMI of 30 kg/m^2^ or higher was the criteria for classifying individuals as obese, with BMI calculated by dividing weight in kilograms by height squared in meters (kg/m^2^) (3). As demonstrated in Figure 1, participants were included at baseline based on the subsequent standards:(1) At least eighteen years of age; (2) Without cancer diseases or being pregnant at baseline; (3) Body mass index (BMI) ≥30 kg/m^2^; (4) Having complete follow-up, BMI, and blood lipid profiles data. Besides, the National Center for Health Statistics’ Ethics Committee authorized the protocols and procedures for the study.

### Exposure and outcome variables

The lipid profile of the fasting blood samples from the participants in this study were used to compute NHHR levels. Non-HDL-C levels were calculated by subtracting HDL-C from TC, and NHHR was calculated by dividing Non-HDL-C by HDL-C (21).

The endpoints of this study involved all-cause mortality and cardiovascular mortality. Death records were obtained from the publicly accessible linked mortality files, encompassing mortality-related variables exclusive to adults. The National Death Index (NDI) offered details about the survival condition and causes of death for the surveyed persons, with data recorded up to December 31, 2019.All-cause mortality was defined as death from any cause. Cardiovascular mortality consisted of deaths caused by CVD or cerebrovascular diseases (22).

### Covariates

Utilizing data information derived from questionnaire, laboratory examinations and physical examinations conducted in the NHANES. We gathered information on age, sex, race, poverty-income ratio, education level, smoking and drinking habit through questionnaire interviews. There were five categories for race: Mexican American, Non-Hispanic Black, Non-Hispanic White, Other Hispanic, and Other Race. The Poverty-Income Ratio (PIR) was calculated as the ratio of household income to the poverty threshold. Three categories were used to categorize educational attainment: above high school, below high school, and high school.

Participants were grouped into two categories: smokers and non-smokers, depending on their answer to a question about smoking at least 100 cigarettes in their lifetime. Additionally, participants were classified as drinkers or non-drinkers based on whether they had ever had more than 12 glasses of alcohol annually. Information on serum biochemical indicator [serum uric acid (SUA), fasting blood glucose (FBG), albumin (ALB), TC, TG, HDL-C, LDL-C] were collected through laboratory examinations. Comorbidities [hypertension, coronary heart disease (CHD), myocardial infarction, heart failure, stroke, hyperlipidemia, gout, diabetes and chronic kidney disease (CKD)], and medication usage (hypertensive medications, lipid-lowering, and diabetic medications) were also considered and included at baseline. Measurements of waist circumference and BMI were taken in accordance with a standardized procedure.

The diagnosis of hypertension was established if any of the following conditions were met: (1) SBP ≥ 140 mmHg and/or DBP ≥ 90 mmHg; (2) Currently taking antihypertensive medication; (3) Acknowledgement of a history of hypertension in a questionnaire; (4) Diagnosed by a clinician (23). Similarly, a history of CHD was diagnosed if the questionnaire responses acknowledge any of the following conditions: angina, coronary heart disease, or heart attack, in which case the individual was classified as having CHD. Hyperlipidemia was defined as TC ≥ 200 mg/dl, TG ≥ 150 mg/dl, LDL-C ≥ 130 mg/dl, or HDL-C ≤ 50 mg/dl for women and ≤40 mg/dl for men, or using lipid-lowering drugs (24). Diabetes mellitus was identified based on FBG ≥ 126 mg/dl, self-reported history of diabetes, hemoglobin A1c levels ≥6.5%, or use of taking diabetes pills (25). Calculations of estimated glomerular filtration rate (eGFR) were performed using the chronic kidney disease epidemiology collaboration (CKD-EPI) equation (26). CKD was defined as participants having an eGFR < 60 ml/min/1.73 m^2^. Other medical history information is obtained through questionnaires.

### Statistical analysis

Proper sampling weights were applied to reconstruct the data representative of the US civilian non-institutionalized population in order to reflect the complex survey methodology of NHANES. According to NHHR concentrations, participants were classified into four groups: 0%–25%, 25%–50%, 50%–75% and 75%–100% (Q1–Q4). Continuous variables were assessed for normality using the Kolmogorov–Smirnov test. Normal distributions were represented as the mean ± standard deviation (mean ± SD), while non-normal variables were denoted as the median (25th percentile, 75th percentile), using the Wilcoxon rank-sum test for inter-group comparisons. Categorical variables were represented as frequencies and weighted percentages, and comparisons were made using chi-square tests.

Multivariate Cox proportional hazards regression models have been applied to explore the linear relationship between NHHR concentration and all-cause mortality and cardiovascular mortality. To investigate any possible nonlinear link between the endpoints and NHHR, Restricted cubic spline (RCS) analysis was used for flexible modeling and identifying a threshold point of NHHR for mortality. Based on these identified thresholds, we performed segmented multivariate Cox regression and subgroup analysis to further examine the relationship between NHHR concentration and mortality risk. Model 1 adjusted for age, gender; Model 2 adjusted for age, sex, race, education level, poverty-income ratio, BMI and waist circumference; Model 3 further adjusted the history of diseases (diabetes, hypertension, gout, CHD, stroke, myocardial infarction, heart failure, CKD) and individual medication history (lipid-lowering drugs, antidiabetic drugs and antihypertensive drugs) based on model 2. We further conducted stratified analyses by age, sex, smoking, drinking, hypertension,diabetes and BMI.Moreover, multiple sensitivity analyses were carried out to evaluate how reliable the findings were. Missing covariates were imputed using the random forest method, which effectively handles missing data by identifying variable types and accounting for collinearity among predictors (27). The “missForest” package was employed for this imputation process. For all statistical analyses, R version 4.3.1 (R Foundation for Statistical Computing, Vienna, Austria) was utilized, and statistical significance was established applying a cut-off of *P* < 0.05.

## Results

### Demographic characteristics demonstrated at baseline

Table 1 presented the baseline characteristics of 7,504 subjects, 4,260 of whom were female, with an average age of 46 years. According to NHHR concentrations (Q1: 0.28–2.23 mmol/L, Q2: 2.24–2.95 mmol/L, Q3: 2.96–3.85 mmol/L and Q4: 3.86–25.81 mmol/L), individuals in the higher NHHR group tended to be younger and predominantly male. Additionally, those in the highest group exhibited increased FBG, SUA, waist circumference, TG, TC, LDL-C, Non-HDL-C, eGFR, albumin and higher rates of smoking, drinking and hyperlipidemia (*p* < 0.05). There were 318 (4.24%) cardiovascular deaths and 866 (11.54%) all-cause deaths over the course of the median follow-up period of 119 months.

**Table 1:** Baseline characteristics of participants stratified by the NHHR concentrations.

Characteristics	Overall	NHHR				*p* value
		Q1, (0.28, 2.23)	Q2, (2.24, 2.95)	Q3, (2.96, 3.85)	Q4, (3.86,25.81)	
*N* (%)	7,504	1,877	1,875	1,876	1,876	
Age, years	46 (34, 58)	48 (33, 62)	48 (35, 60)	46 (33, 57)	44 (34, 55)	<0.001
Sex, (%)						<0.001
Female	4,260 (54%)	1,360 (71%)	1,225 (64%)	952 (47%)	723 (37%)	
Male	3,244 (46%)	517 (29%)	650 (36%)	924 (53%)	1,153 (63%)	
Race, (%)						<0.001
Mexican American	1,549 (10%)	317 (9.0%)	375 (10%)	431 (11%)	426 (11%)	
Non-Hispanic Black	2,013 (15%)	711 (23%)	531 (15%)	444 (13%)	327 (9.5%)	
Non-Hispanic White	2,882 (64%)	592 (58%)	721 (65%)	728 (66%)	841 (68%)	
Other Hispanic	680 (5.8%)	170 (6.3%)	164 (5.6%)	172 (5.4%)	174 (6.1%)	
Other Race	380 (4.7%)	87 (4.0%)	84 (3.9%)	101 (5.2%)	108 (5.7%)	
Education Level, (%)						0.002
Above high school	3,586 (55%)	969 (59%)	925 (57%)	885 (56%)	807 (50%)	
High school	1,777 (26%)	403 (23%)	447 (26%)	468 (26%)	459 (28%)	
Below high school	2,141 (19%)	505 (19%)	503 (17%)	523 (18%)	610 (22%)	
Smoking habit, (%)	3,193 (45%)	708 (40%)	780 (43%)	805 (45%)	900 (49%)	<0.001
Drinking behavior, (%)	5,113 (73%)	1,220 (71%)	1,230 (71%)	1,296 (75%)	1,367 (76%)	0.006
poverty-income ratio	2.64 (1.44, 4.44)	2.53 (1.44, 4.40)	2.68 (1.44, 4.41)	2.73 (1.47, 4.61)	2.56 (1.36, 4.28)	0.306
BMI, kg/m^2^	34.2 (31.8, 38.4)	34.0 (31.7, 38.4)	34.5 (31.9, 38.9)	34.1 (31.8, 38.3)	34.2 (31.8, 38.2)	0.189
Waist circumference, cm	112 (106, 121)	110 (103, 119)	112 (104, 122)	113 (106, 122)	113 (107, 122)	<0.001
SUA, IU/L	345 (292, 405)	315 (268, 375)	333 (280, 381)	357 (303, 416)	375 (321, 428)	<0.001
Albumin, g/L	41.0 (39.0, 44.0)	41.0 (38.2, 43.0)	41.0 (39.0, 43.0)	42.0 (40.0, 44.0)	42.0 (40.0, 44.0)	<0.001
TC, mmol/L	4.94 (4.29, 5.64)	4.24 (3.73, 4.86)	4.73 (4.16, 5.30)	5.04 (4.50, 5.64)	5.61 (4.99, 6.31)	<0.001
TG, mmol/L	1.39 (0.97, 1.95)	0.88 (0.67, 1.23)	1.17 (0.91, 1.58)	1.51 (1.16, 1.96)	2.03 (1.57, 2.66)	<0.001
LDL, mmol/L	3.00 (2.43, 3.59)	2.28 (1.86, 2.72)	2.84 (2.41, 3.26)	3.16 (2.69, 3.65)	3.65 (3.13, 4.27)	<0.001
HDL, mmol/L	1.22 (1.03, 1.42)	1.53 (1.32, 1.78)	1.32 (1.16, 1.47)	1.16 (1.03, 1.29)	0.98 (0.85, 1.09)	<0.001
None-HDL-C, mmol/L	3.69 (3.06, 4.37)	2.74 (2.32, 3.16)	3.40 (3.02, 3.83)	3.88 (3.47, 4.36)	4.63 (4.09, 5.25)	<0.001
NHHR, mmol/L	3.05 (2.32, 3.93)	1.84 (1.57, 2.05)	2.61 (2.42, 2.77)	3.36 (3.15, 3.58)	4.58 (4.19, 5.21)	<0.001
FBG, mg/L	102 (95, 114)	101 (93, 112)	102 (95, 114)	103 (96, 112)	104 (96, 115)	<0.001
eGFR, ml/min/1.73 m^2^	100 (86, 112)	99 (82, 111)	98 (84, 111)	101 (87, 113)	103 (89, 114)	<0.001
Diabetes Mellitus, (%)	1,780 (20%)	471 (21%)	451 (21%)	415 (18%)	443 (20%)	0.165
CKD, (%)	463 (4.4%)	153 (7.4%)	116 (4.0%)	103 (3.7%)	91 (3.2%)	<0.001
Hypertension, (%)	3,650 (46%)	966 (47%)	933 (47%)	875 (45%)	876 (45%)	0.491
Hyperlipidemia, (%)	6,130 (82%)	1,009 (54%)	1,484 (77%)	1,761 (94%)	1,876 (100%)	<0.001
CHD, (%)	569 (6.6%)	159 (7.6%)	157 (7.3%)	126 (5.8%)	127 (6.0%)	0.207
MI, (%)	350 (3.9%)	99 (4.2%)	88 (3.7%)	75 (3.7%)	88 (4.0%)	0.901
HF, (%)	279 (2.8%)	78 (3.1%)	67 (2.4%)	71 (3.1%)	63 (2.8%)	0.743
Stroke, (%)	275 (2.9%)	87 (3.4%)	59 (2.2%)	63 (3.2%)	66 (3.0%)	0.333
Gout, (%)	305 (3.6%)	76 (3.6%)	80 (3.8%)	78 (3.6%)	71 (3.5%)	0.969
Lipid-lowering drugs, (%)	1,828 (24%)	487 (26%)	454 (23%)	441 (23%)	446 (23%)	0.298
Antidiabetic drugs, (%)	507 (5.1%)	165 (6.8%)	131 (4.7%)	111 (5.4%)	100 (3.6%)	<0.001
Antihypertensive drugs, (%)	2,842 (35%)	802 (39%)	756 (38%)	663 (34%)	621 (32%)	0.002
All-cause death, (%)	866 (9.2%)	227 (10%)	215 (8.2%)	197 (8.6%)	227 (10%)	0.178
Cardiovascular death, (%)	318 (3.3%)	88 (4.2%)	87 (3.1%)	71 (2.9%)	72 (3.2%)	0.211

Data presented median (p25, p75) for continuous and *n* (%) for categorical. Wilcoxon rank-sum test for complex survey samples; Chi-squared test with Rao and Scott's second-order correction.

NHHR, non-high-density lipoprotein cholesterol to high-density; BMI, body mass index; SUA, serum uric acid; HDL-C, high-density lipoprotein cholesterol; TC, total cholesterol; Non-HDL-C, non-high-density lipoprotein cholesterol; LDL-C, low-density lipoprotein cholesterol; TG, triglyceride; FBG, fasting blood glucose; eGFR, estimated glomerular filtration; CKD, chronic kidney disease; CHD, coronary heart disease; MI, myocardial infarction; HF, heart failure.

### Relationship between the NHHR and the mortality risk

In the entire obese population, NHHR, when included as a continuous variable in the three regression model, failed to demonstrate a statistically significant correlation with either cardiovascular or all-cause mortality (Table 2).

**Table 2:** Associations of NHHR levels with all-cause and cardiovascular mortality in patients with obesity.

Characteristic	Model 1			Model 2			Model 3		
	HR	95% CI	*p* value	HR	95% CI	*p* value	HR	95% CI	*p* value
All-cause mortality									
NHHR per 1 mmol/L increase	1.04	0.95, 1.14	0.400	1.02	0.92, 1.12	0.717	1.02	0.93, 1.13	0.618
Cardiovascular mortality									
NHHR per 1 mmol/L increase	0.99	0.86, 1.15	0.894	0.98	0.84, 1.15	0.823	0.99	0.86, 1.15	0.934

HR, hazard ratio; CI, confidence interval; NHHR, non-high-density lipoprotein cholesterol to high-density.

Model 1: adjusted for age, sex.

Model 2: model 1 + race, education level, poverty-income ratio, body mass index and waist circumference.

Model 3: model 2 + diabetes, hypertension, chronic kidney disease, gout, coronary heart disease, stroke, myocardial infarction, heart failure, lipid-lowering drugs, antidiabetic drugs and antihypertensive drugs.

**Figure 2:**
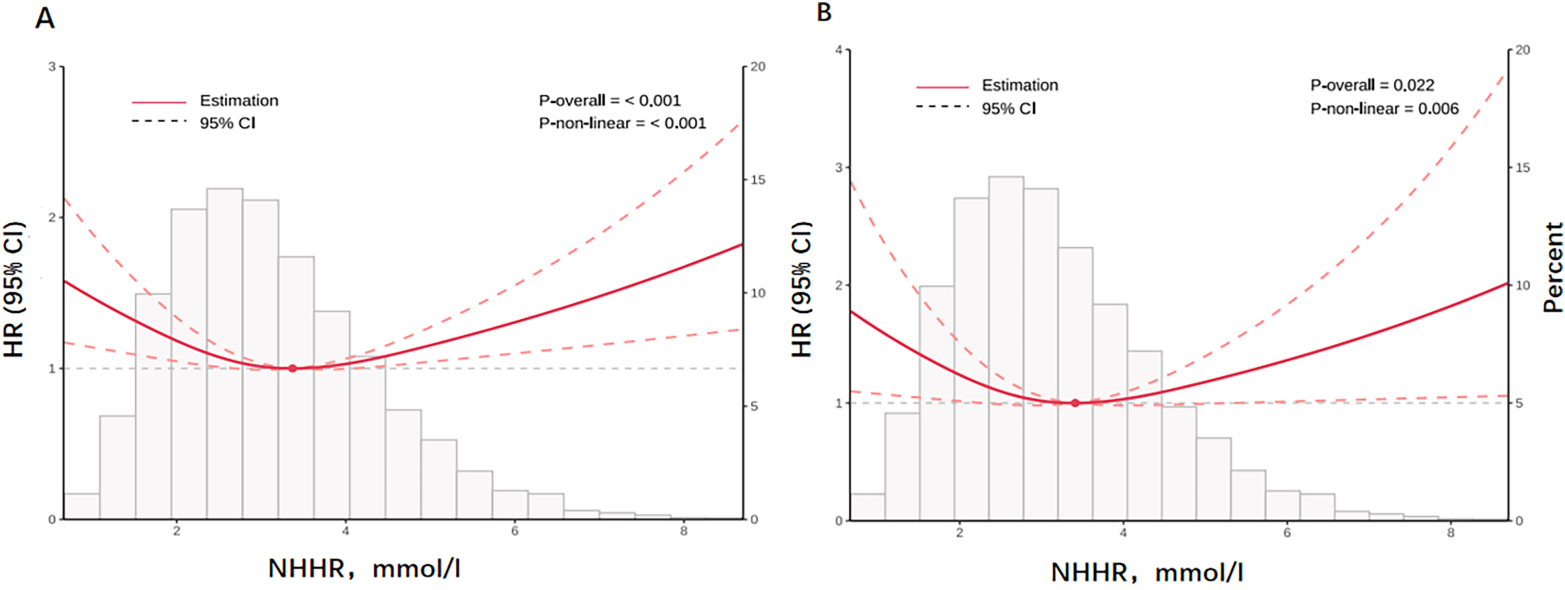
The figure illustrates the nonlinear relationship between NHHR and all-cause **(A)** and cardiovascular mortality **(B)** on a continuous scale. Histogram of the frequency distribution of the study cohort displayed in the background. Analyses were adjusted for confounding factors based model3. Solid red lines are multivariable adjusted hazard ratios, with dashed red lines representing 95% confidence intervals.

A U-shaped association between NHHR and the risk of all-cause (P for nonlinear <0.001, Figure 2A) and cardiovascular mortality (P for nonlinear = 0.006, Figure 2B) was shown by the restricted cubic spline (RCS) curves. A cut-off point for NHHR was observed in our study. The lowest risk of all-cause and cardiovascular death was linked to NHHR concentrations of 3.369 mmol/L and 3.409 mmol/L, respectively (Figure 2).

As shown in Table 3, when NHHR was incorporated into the final fully adjusted model as a continuous variable, we observed that at NHHR concentrations below the cut-off value, an increase in each unit of NHHR level was significantly negatively correlated with all-cause mortality (HR = 0.82, 95% CI: 0.67–1.00) and cardiovascular mortality (HR = 0.68, 95% CI: 0.49–0.94); conversely, at NHHR concentrations greater than the cut-off value, relatively higher levels of NHHR were significantly positively correlated with all-cause mortality (HR = 1.13, 95% CI: 1.01–1.28) and cardiovascular mortality (HR = 1.18, 95% CI: 1.02–1.36).

**Table 3:** Explore the relationship between NHHR and mortality using segmented Cox regression.

Characteristic	Model 1			Model 2			Model 3		
	HR	95% CI	*p* value	HR	95% CI	*p* value	HR	95% CI	*p* value
All-cause mortality									
NHHR < 3.369	0.83	0.68, 1.00	0.051	0.79	0.65, 0.96	0.020	0.82	0.66, 1.00	0.050
NHHR > 3.369	1.14	1.03, 1.26	0.011	1.14	1.02, 1.28	0.021	1.13	1.01,1.28	0.035
CVD mortality									
NHHR < 3.409	0.66	0.49, 0.90	0.008	0.66	0.48, 0.91	0.012	0.68	0.49,0.94	0.021
NHHR > 3.409	1.16	1.00, 1.33	0.048	1.19	1.02, 1.38	0.026	1.18	1.02,1.36	0.026

HR, hazard ratio; CI, confidence interval; NHHR, non-high-density lipoprotein cholesterol to high-density.

Model 1: adjusted for age, sex.

Model 2: model 1 + race, education level, poverty-income ratio, body mass index and waist circumference.

Model 3: model 2 + diabetes, hypertension, chronic kidney disease, gout, coronary heart disease, stroke, myocardial infarction, heart failure, lipid-lowering drugs, antidiabetic drugs and antihypertensive drugs.

### Subgroups analysis

Supplementary Figure S1 presented the results of the segmented subgroup analysis and interaction tests based on the cut-off points between the NHHR and mortality. No significant interactions were found between the various subgroups.

When NHHR levels were below the threshold, significant negative associations with mortality were observed among people who were sixty years of age or older, males, and smokers. Individuals with a habit of alcohol consumption (HR = 0.75, 95% CI: 0.62–0.91) and with a history of hypertension (HR = 0.81, 95% CI: 0.68–0.96) also showed a significant negative association with all-cause mortality.

In contrast, when NHHR levels were exceeded the threshold, individuals with no smoking history (HR = 1.25, 95% CI: 1.04–1.51) and no drinking habits (HR = 1.21, 95% CI: 1.00–1.45) significantly increase the chance of dying from all causes; those under the age of 60 (HR = 1.23, 95% CI: 1.03–1.47) significantly raise the chance of dying from cardiovascular disease.

### Sensitivity analysis

To confirm the credibility of the U-shaped connection between NHHR and mortality, we conducted several sensitivity analyses. First, we excluded individuals over the age of 65 and those who experienced events within 1 year of follow-up to minimize the impact of severe acute illnesses on the outcomes. Second, considering the impact of lipid-lowering medication on blood lipid levels, those taking medications to decrease cholesterol at baseline were not included. Third, adjusted for age, gender, race, education levels, BMI, smoking, alcohol use, waist circumference, diabetes, hypertension, gout, CHD, stroke, myocardial infarction, heart failure, CKD, lipid-lowering drugs, antidiabetic drugs and antihypertensive drugs to assess the connection between NHHR levels and mortality. We found that the results were similar to previous studies (Supplementary Figure S2).

## Discussion

In this study, we found that in obese adults, NHHR revealed a U-shaped relationship with both cardiovascular and all-cause death. For all-cause and cardiovascular mortality, the lowest risk was observed at cut-off point of 3.369 and 3.409 mmol/L, respectively. Relatively greater or lower NHHR concentrations were associated with higher likelihood of death.

A key observation from our research was that individuals with increased NHHR levels face a greater risk of mortality. Consistent with our study findings, numerous previous studies have demonstrated an independent association between the NHHR and cardiovascular risk, establishing NHHR as a valuable lipid parameter for assessing the risk of CVD in general population (12, 28, 29). Nevertheless, no prior research has investigated the connection between NHHR levels and the mortality risk in the obese adults. Obesity significantly increases the risk of CVD and all-cause mortality, in part due to the promotion of dyslipidemia, which is a lipid profile associated with atherosclerosis (7). This particular dyslipidemia pattern, propelled by insulin resistance, is characterized by elevated TG and lowered HDL-C levels. Additionally, there are qualitative abnormalities observed in the LDL particles and HDL particles (30). At the same time, obesity hastens the onset of atherosclerotic alterations via a range of pathways, such as insulin resistance and inflammatory processes (5). It has been established that elevated Non-HDL-C levels are associated with a higher risk of death from cardiovascular and other causes (31–33). Non-HDL-C, defined as the total cholesterol carried by all atherogenic lipoproteins [including LDL-C, triglyceride-rich lipoproteins (TRL), TRL remnants, and lipoprotein a Lp(a)], was found to be a stronger indicator of cardiovascular disease risk than LDL-C in a study from the large-scale Copenhagen General Population Study (34). This implies that elevated levels of NHHR in obese people could play an essential part in enhancing the probability of cardiovascular illnesses thus raising the rates of cardiovascular and overall mortality.

Notably, our study also revealed that lower NHHR levels were associated with increased mortality risk in obese patients. This observation aligns with several previous studies examining lipid parameters and mortality risk. Notably, our previous study on US patients with diabetes or prediabetes also found a U-shaped association between NHHR and all-cause mortality, and an L-shaped association with cardiovascular mortality (13). The current finding of a U-shaped association between NHHR and cardiovascular mortality in the obese population suggests that this nonlinear relationship might be common across populations with metabolic abnormalities. However, it's worth noting that in populations with milder metabolic disorders, such as prediabetes, this relationship may be attenuated or absent, possibly due to insufficient metabolic factor-related mortality burden.

Similar U-shaped relationships between lipid parameters and mortality have been consistently reported in various populations. Cheng et al. demonstrated a U-shaped relationship between Non-HDL-C levels and both cardiovascular and all-cause mortality risk among hypertensive individuals during a 7.7-year follow-up (35). Similarly, the Copenhagen general population study, with a 9.4-year median follow-up, revealed a U-shaped association between LDL-C and all-cause mortality risk, persisting in individuals not taking lipid-lowering drugs (36). Rong et al.'s study of the US population, with a 23.2-year median follow-up, found that extremely low LDL-C levels were associated with increased cardiovascular and all-cause mortality risks (37). Like these studies, our investigation collected lipid parameters at baseline and adjusted for key confounding variables (age, gender, ethnicity, and comorbidities), consistently finding that low baseline NHHR concentrations were associated with higher long-term mortality risk.

The association between low NHHR and increased mortality risk may be explained by several clinical characteristics observed in our study population. Compared to those in the NHHR 50th−75th percentile group, obese individuals with NHHR ≤ 25th percentile were older, had higher usage rates of lipid-lowering, hypoglycemic, and antihypertensive medications, and showed greater prevalence of comorbidities (CKD, hypertension, diabetes mellitus, and CVD). Additionally, these patients exhibited lower blood albumin levels, suggesting compromised nutritional status.

The relationship between low NHHR and increased mortality risk may involve multiple mechanisms. Reports have indicated that low TC can contribute to malnutrition, cachexia, and a significant burden of systemic inflammation, suggesting that the association between low NHHR and poor prognosis may be attributed to frailty. Although we excluded cancer patients at baseline, there might be unidentified non-cardiovascular conditions affecting health outcomes. Obese individuals are particularly susceptible to certain cancers and infections (8, 38), and low NHHR levels may indicate underlying frailty and disease burden. Disease-related malnutrition and chronic inflammatory states could exacerbate the condition in these vulnerable patients.

Furthermore, low NHHR levels may result from either elevated HDL-C or reduced Non-HDL-C. Recent research has shown that elevated HDL-C levels correlate with higher risks of both cardiovascular and all-cause mortality (39–42). This unexpected association may be explained by changes in HDL particle structure and functional properties under inflammatory conditions (30, 43). Given that obesity significantly impacts HDL metabolic enzyme activity and protein composition, monitoring NHHR levels may have clinical significance in obese populations.

The advantages of the study lie in the selection of samples from a nationally representative sampling, along with a long follow-up period, ensuring representativeness. Although LDL-C is the main lipid marker used in current guidelines for evaluating cardiovascular risk, our study indicates that NHHR might offer extra prognostic insights beyond those provided by LDL-C in obese adults.

The following are the study's limitations. First, despite adjusting for covariates, there are still unconsidered confounding factors, such as inflammatory markers and physical activity. Additionally, disease status was obtained through self-reported questionnaires, which may introduce recall bias or result in underdiagnosis. Second, the NHANES dataset only provides baseline lipid measurements, preventing the analysis of longitudinal changes in lipid profiles. This single baseline measurement cannot capture the cumulative lipid exposure from baseline to event occurrence, which is crucial for cardiovascular risk assessment, and may be influenced by dietary habits and lifestyle factors at the time of measurement. Additionally, single measurements are susceptible to measurement error. Future studies using databases with longitudinal lipid measurements would help validate these findings and better understand temporal changes in lipid profiles. Lastly, as the survey sample is selected from the American population, the findings need to be validated in different countries and ethnic backgrounds to assess generalizability. The minimum risk threshold and applicability for specific populations still need to be validated in different settings.

## Conclusion

In obese adults, the NHHR exhibited a U-shaped relationship with cardiovascular and all-cause mortality. Monitoring and managing NHHR levels in obese population may help mitigate the mortality risk.

## Data Availability

The datasets presented in this study can be found in online repositories. The names of the repository/repositories and accession number(s) can be found below: https://www.cdc.gov/nchs/nhanes/?CDC_AAref_Val=https://www.cdc.gov/nchs/nhanes/index.htm.

## Supplementary material

Supplementary Figure S1 Segmented subgroup analysis of the NHHR Segmented subgroup analyses were performed based on respective cut-off points (all-cause mortality: 3.369; cardiovascular mortality: 3.409) to examining the link between NHHR levels and outcomes. Multivariate Cox regression model was adjusted for age, gender, race, education levels, body mass index, waist circumference, diabetes, hypertension, gout, coronary heart disease, stroke, myocardial infarction, heart failure and chronic kidney disease, lipid-lowering drugs, antidiabetic drugs and antihypertensive drugs. **(A)** Association between NHHR and all-cause mortality when NHHR concentration <3.369. **(B)** Association between NHHR and all-cause mortality when NHHR concentration >3.369. **(C)** Association between NHHR and cardiovascular mortality when NHHR concentration <3.409. **(D)** Association between NHHR and cardiovascular mortality when NHHR concentration >3.409.

Supplementary Figure S2 Sensitivity analysis in the study population by restricted cubic spline regressions. **(A)** Exclude age ≥65 and events occurring within 1 year of follow-up. **(B)** Excluding individuals using lipid-lowering medications. **(C)** adjusted for age, gender, race, education levels, body mass index, smoking, alcohol use, waist circumference, diabetes, hypertension, gout, coronary heart disease, stroke, myocardial infarction, heart failure and chronic kidney disease, lipid-lowering drugs, antidiabetic drugs and antihypertensive drugs.
