# Supplementary figures and images for: U-shaped association between the non-high-density lipoprotein cholesterol to high-density lipoprotein cholesterol ratio and mortality risk in obese adults: evidence from NHANES 1999–2018
Source: Front Cardiovasc Med. 2025 Jan 10;11:1524465. doi: 10.3389/fcvm.2024.1524465 (PMC11759299; doi:10.3389/fcvm.2024.1524465)

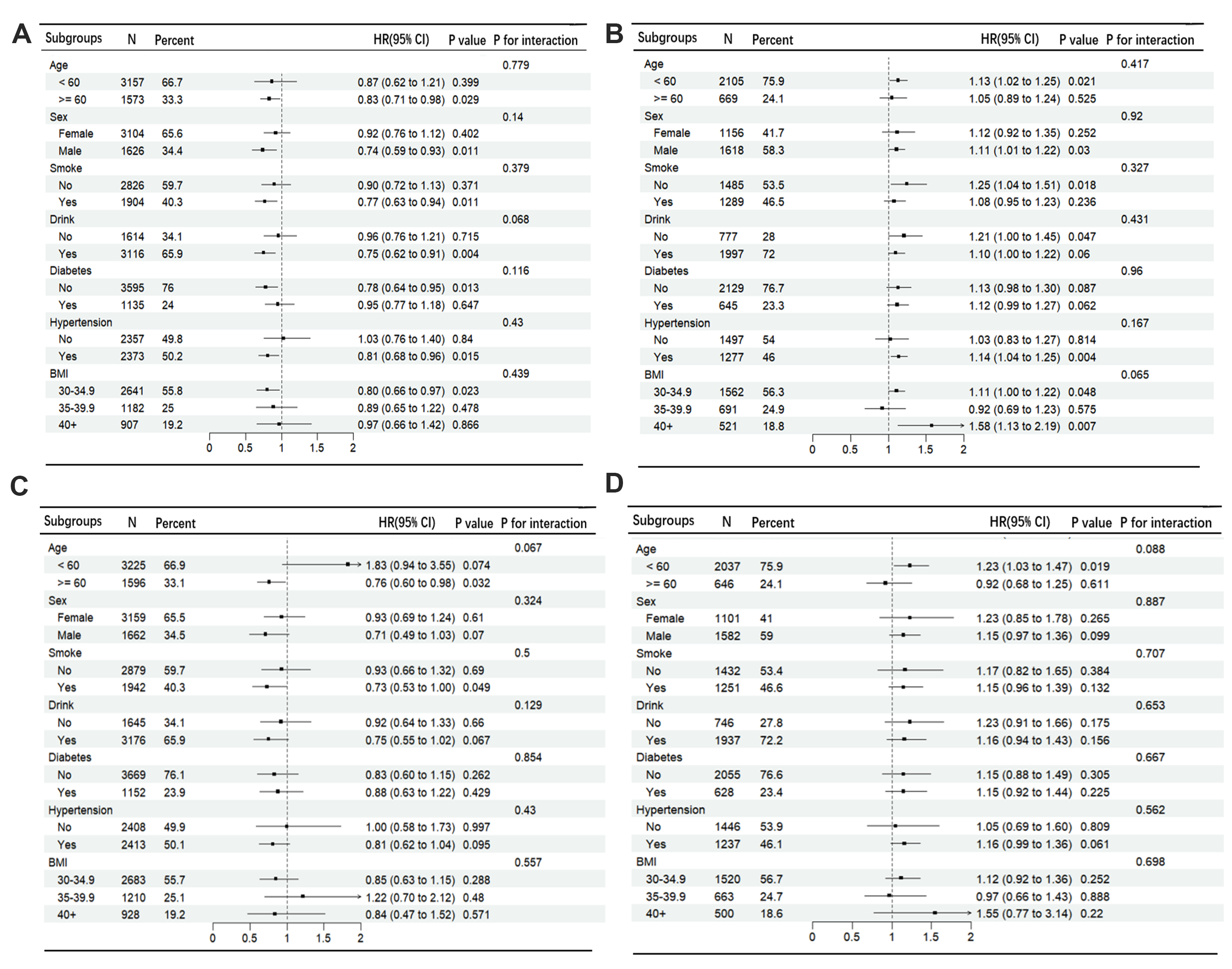

Supplement: Supplementary Figure S1 — Segmented subgroup analysis of the NHHR Segmented subgroup analyses were performed based on respective cut-off points (all-cause mortality: 3.369; cardiovascular mortality: 3.409) to examining the link between NHHR levels and outcomes. Multivariate Cox regression model was adjusted for age, gender, race, education levels, body mass index, waist circumference, diabetes, hypertension, gout, coronary heart disease, stroke, myocardial infarction, heart failure and chronic kidney disease, lipid-lowering drugs, antidiabetic drugs and antihypertensive drugs. (A) Association between NHHR and all-cause mortality when NHHR concentration <3.369. (B) Association between NHHR and all-cause mortality when NHHR concentration >3.369. (C) Association between NHHR and cardiovascular mortality when NHHR concentration <3.409. (D) Association between NHHR and cardiovascular mortality when NHHR concentration >3.409. [file Image1.tif]

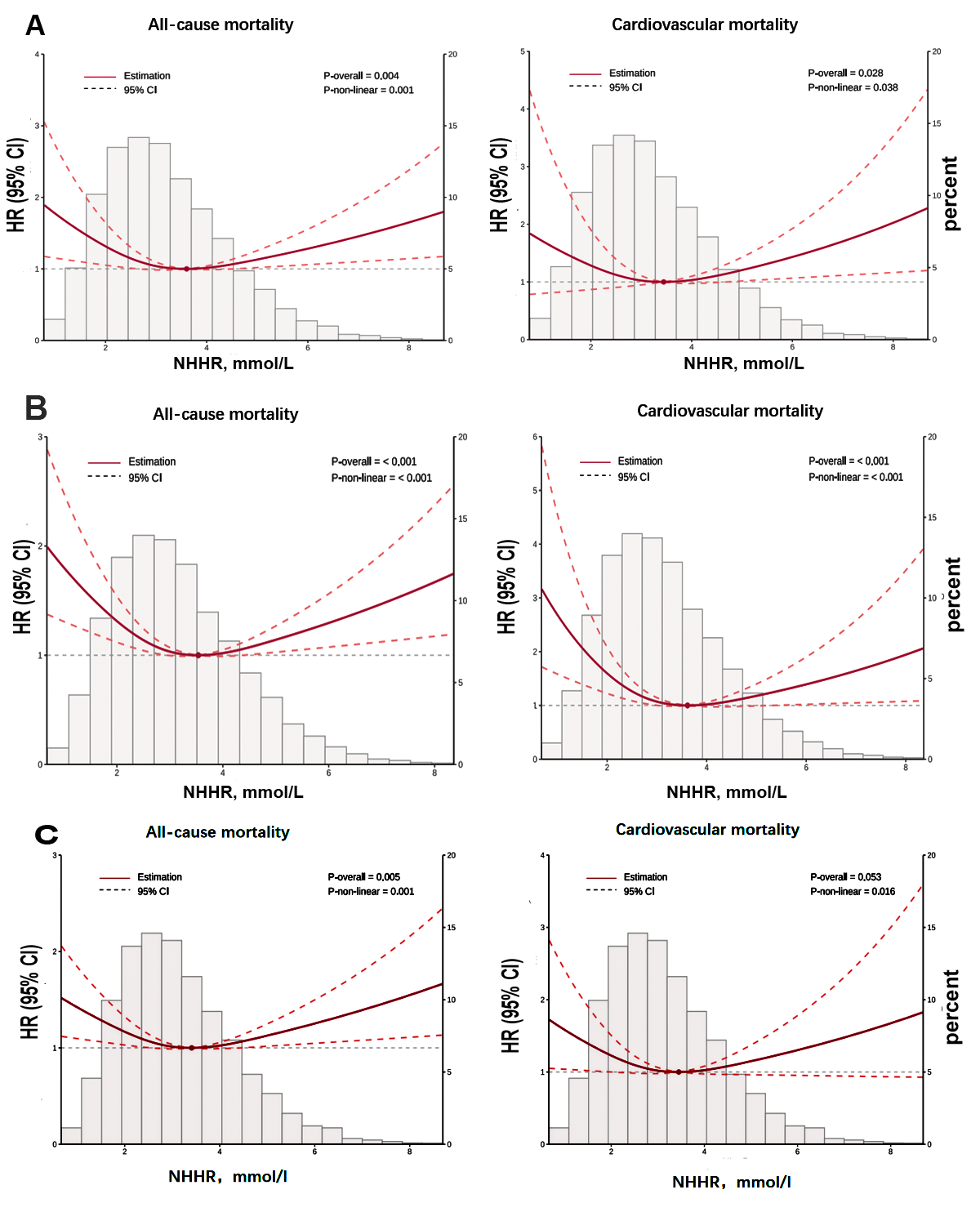

Supplement: Supplementary Figure S2 — Sensitivity analysis in the study population by restricted cubic spline regressions. (A) Exclude age ≥65 and events occurring within 1 year of follow-up. (B) Excluding individuals using lipid-lowering medications. (C) adjusted for age, gender, race, education levels, body mass index, smoking, alcohol use, waist circumference, diabetes, hypertension, gout, coronary heart disease, stroke, myocardial infarction, heart failure and chronic kidney disease, lipid-lowering drugs, antidiabetic drugs and antihypertensive drugs. [file Image2.tif]
